# Synthesis and Pharmacological Evaluation of Novel Schiff Base Analogues of 3-(4-amino) Phenylimino) 5-fluoroindolin-2-one

**DOI:** 10.4103/0975-1483.63162

**Published:** 2010

**Authors:** R Nirmal, CR Prakash, K Meenakshi, P Shanmugapandiyan

**Affiliations:** *Department of Pharmaceutical Chemistry, Balaji Institute of Pharmacy, Laknepally (V), Narsampet (M), Warangal, Andhra Pradesh – 506 331, India*; *DCRM Pharmacy College, Inkollu (V), Prakasam (D), Andhra Pradesh– 523 167, India*

**Keywords:** Analgesic, anti-inflammatory, 5-fluoroindolin-2-one

## Abstract

In our study, a series of novel 3-(4-(benzylideneamino) phenylimino) 4-fluoroindolin-2-one derivatives were synthesized and characterized by spectral (I.R, ^1^H NMR, mass) and elemental analysis. The title compounds (N_1_-N_10_) were evaluated for analgesic, anti-inflammatory, and ulcerogenic index activities. Results displayed that compound N_3_ exhibited significant analgesic activity. Among the title compounds studied, N_2_, N_3_, and N_8_ exhibited significant anti- inflammatory activity comparable to reference standard diclofenac sodium. Interestingly, the test compounds showed only mild ulcerogenic side effect when compared to aspirin.

## INTRODUCTION

Compounds with the structure of -C=N- (azomethine group) are known as Schiff bases, which are usually synthesized from the condensation of primary amines and active carbonyl groups. Schiff bases are important class of compounds in medicinal field. Ample studies demonstrated the biological applications including antibacterial,[1–6] antifungal,[3–6] and antitumor activities.[7,8] Similarly, indole derivatives are known to possess a variety of biological activities such as CNS depressant, anticancerous, antimicrobial, antihistaminic, anticonvulsants, and many others.[9] Many naturally occurring products contain the indole skeleton as a backbone of their structural frameworks.[10]

Based on the above-mentioned properties of Schiff bases and indole analogous, in our present study we report their synthesis, spectroscopic characterization and the pharmacological profile of Schiff base analogous 5-fluoroindolin 2-one. The synthesized compounds were screened for their analgesic, anti-inflammatory, and ulcerogenic index activities.

## MATERIALS AND METHODS

### Chemistry

Melting points (mp) were taken in open capillaries on Thomas Hoover melting point apparatus and are uncorrected. The IR spectra were recorded in film or in potassium bromide disks on a Perkin-Elmer 398 spectrometer. The ^1^H spectra were recorded on a DPX-300 MHz Bruker FT-NMR spectrometer. The chemical shifts were reported as parts per million (δ ppm) tetramethylsilane (TMS) as an internal standard. Mass spectra were obtained on a JEOL-SX-102. instrument using fast atom bombardment (FAB positive). Elemental analysis was performed on a Perkin-Elmer 2400 C, H, N analyzer and values were within the acceptable limits of the calculated values. The progress of the reaction was monitored on readymade silica gel plates (Merck) using chloroform/methanol (9:1) as a solvent system. Iodine was used as a developing agent. Spectral data (IR, ^1^H NMR and mass spectra) confirmed the structures of the synthesized compounds and the purity of these compounds was ascertained by microanalysis. Elemental (C, H, and N) analysis indicated that the calculated and observed values were within the acceptable limits (±0.4%). All chemicals and reagents were obtained from Aldrich (USA), Lancaster (UK), or Spectrochem Pvt. Ltd (India) and were used without further purification.

### Synthesis of 5- fluoro imesatins (4)

Chloral hydrate (0.054 moles) in water (120 ml) was prepared. To this 4-fluoro aniline (1) (0.158 mole) and sodium sulfate (0.05mol) was added dropwise for 30 min with stirring and finally aqueous hydroxylamine hydrochloride (50 ml) was added gradually keeping the reaction mixture was heated in about 45 min. During the heating period, some crystals of 4-fluoro isonitrosoacetanilide (2) separates out. The solid obtained was filtered, washed with water, dried and recrystallized from ethanol. Concentrated H_2_SO_4_ was added gradually to the resulting compound (2) and boiled for 10 min. The reaction mixture was then poured into ice water and the 5-fluoroisatin (3) filtered and purified. 4-amino aniline (0.01 moles) and the above prepared 5-fluoroisatin (3) were dissolved in warm ethanol and refluxed for 30 min. After standing for approximately 24 h at room temperature, the 5-fluoroimesatins (4) were separated by filtration and recrystallized from warm ethanol. Yield = 83%, mp 260-262 °C. IR (KBr) cm^-1^: 3220 (NH), 1675 (C = O); ^1^H NMR (CDCl_3_): δ 7.15-8.74 (m, 8H, Ar-H), 10.71 (s, 1H, NH); MS (m/z) 237 (M^+^). Anal. Calcd for C_14_H_11_N_3_O: C, 70.87, H, 4.67; N, 17.71. Found: C, 70.83, H, 4.66, N, 17.72.

### General synthetic procedure for compounds (N_1_- N_10_)

**Scheme 1:**
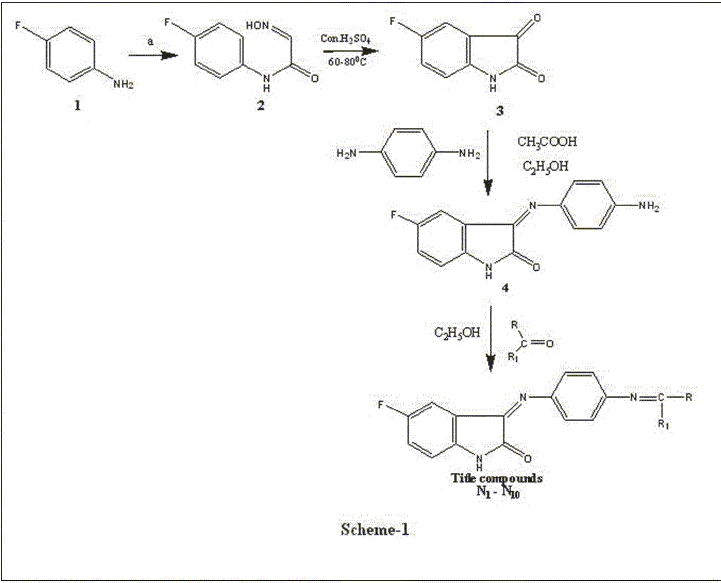
Synthetic scheme for the title compounds N_1_ – N_10_

A mixture of 5-fluoro imesatin (4) (0.01 moles) and 0.01 moles of appropriate ketone/aldehyde was dissolved in 30 ml of ethanol [Scheme 1]. Then refluxed for 8 h and kept aside. The solid obtained was filtered, washed with water, dried under high vacuum, and recrystallized from chloroform/benzene (25:75) mixture. The physical and spectral data of the synthesized compounds are presented in Tables 1 and 2.

**Table 1:**
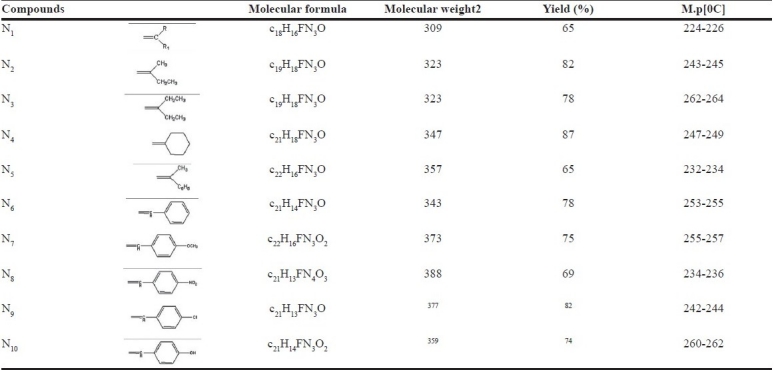
Physicochemical data of 3-(4-amino) phenylimino)-5-fluoroindolin-2-one (N_1_-N_10_)

**Table 2:** NMR and IR spectral data of the novel synthesized compounds (N_1_-N_10_)

Compound Code	IR NH	(cm^-1^) C=O	(KBr) C=N	1H NMR (CDCl_3_) δ ppm
N_1_	3290	1687	1614	1.2-1.3 (q, 2H, CH_2_CH_3_), 1.5-1.6 (t, 3H, CH_2_CH_3_), 7.3-8.0 (m, 7H, Ar-H), 8.3 (s, 1H, NH).
N_2_	3288	1675	1611	1.0-1.2 (m, 4H, (CH_2_CH_3_)2), 1.7-1.9 (m, 6H, (CH_2_CH_3_)2), 7.4-8.1 (m, 7H, Ar-H), 8.2 (s, 1H, NH).
N_3_	3275	1685	1610	0.9-1.0 (t, 2H, CH_2_CH_2_CH_3_), 1.2-1.4 (sext, 2H, CH_2_CH_2_CH_3_),1.7-1.8 (t, 3H, CH_2_CH_2_CH_3_), 2.4 (s, 3H, CH_3_), 7.0-7.7 (m, 8H, Ar-H), 8.2 (s, 1H, NH).
N_4_	3289	1688	1615	1.1-1.9 (m, 10H, cyclohexanyl), 7.0-7.7 (m, 7H, Ar-H), 8.5 (s, 1H, NH).
N_5_	3278	1682	1617	1.5 (s, 3H, CH_3_), 7.1-8.3 (m, 12H, Ar-H), 8.6 (s, 1H, NH).
N_6_	3292	1685	1615	6.2 (s, 1H, CH), 7.1-8.2 (m, 12H, Ar-H), 8.5 (s, 1H, NH).
N_7_	3286	1676	1612	2.7-2.8 (s, 3H, OCH_3_), 6.1 (s, 1H, CH), 7.0-8.2 (m, 11H, Ar-H), 8.6 (s, 1H, NH).
N_8_	3292	1680	1619	6.4 (s, 1H, CH), 7.2-8.3 (m, 11H, Ar-H), 8.7 (s, 1H, NH).
N_9_	3294	1668	1610	6.2 (s, 1H, CH), 7.0-8.1 (m, 11H, Ar-H), 8.3 (s, 1H, NH).
N_10_	3287	1679	1614	5.7 (s, 1H, Ar-OH). 6.4 (s, 1H, CH), 7.2-8.3 (m, 11H, Ar-H), 8.5 (s, 1H, NH).

### Pharmacology

The synthesized compounds were evaluated for analgesic, anti-inflammatory, and ulcerogenic index. One-way analysis of variance (ANOVA) was performed to ascertain the significance of all the exhibited activities. The test compounds and the standard drugs were administered in the form of a suspension (1% carboxymethyl cellulose as a vehicle) by oral route for analgesic and anti-inflammatory but for ulcerogenicity studies by intraperitoneally as suspension in 10% v/v Tween-20. Each group consisted of six animals. The animals were procured from the C. L. Baid Metha College of pharmacy, Chennai, and were maintained in colony cages at 25 ±2°C, relative humidity of 45-55%, under a 12-h light and dark cycle; they were fed standard animal feed. All the animals were acclimatized for a week before use. The Institutional Animal Ethics Committee approved the protocol adopted for the experimentation of animals.

### Analgesic activity

The analgesic activity was performed by tail-flick technique using Wistar albino mice (25-35 g) of either sex selected by the random sampling technique.[13,14] Diclofenac sodium at a dose level of 10 mg/kg and 20 mg/kg was administered orally as reference drug for comparison. The test compounds at two dose levels (10, 20 mg/kg) were administered orally. The reaction time was recorded at 30 min, 1, 2, and 3 h after the treatment, and cut-off time was 10 s. The percent analgesic activity (PAA) was calculated by the following formula, where T_1_ is the reaction time (s) before treatment, and T_2_ is the reaction time (s) after treatment.

$$\mathrm{PAA}=\left\{\frac{T_{1}\;-T_{2}}{10-T_{1}}\times 100\right\}$$

### Anti-inflammatory activity

Anti-inflammatory activity was evaluated by carrageenan-induced paw edema test in rats.[15] Diclofenac sodium 10, 20 mg/kg was administered as a standard drug for comparison. The test compounds were administered at two dose levels (10 mg/kg, 20 mg/kg). The paw volumes were measured using the mercury displacement technique with the help of a plethysmograph (Model PLYAN; Buxco, USA) immediately before and 30 min, 1, 2, and 3 h after carrageenan injection. The percent inhibition of paw edema was calculated using the following formula.

$$\mathrm{Percent}\;\mathrm{inhibition}\;I\;=\left[\;100\;\left[1-\;(a-x)\right]\;/\;(b-y)\right]$$

Where *x* is the mean paw volume of rats before the administration of carrageenan and test compounds or reference compound (test group), *a* is the mean paw volume of rats after the administration of carrageenan in the test group (drug treated), *b* is the mean paw volume of rats after the administration of carrageenan in the control group, *y* is the mean paw volume of rats before the administration of carrageenan in the control group.

### Evaluation of ulcerogenicity index

Ulceration in rats was induced as described by Goyal *et al*. Albino rats of Wistar strain weighing 150-200 g of either sex were divided into various groups each of six animals.[16] Control group of animals was administered only with 10% v/v Tween 80 suspension intraperitonially. One group was administered with aspirin (German Remedies) intraperitoneally at a dose of 200 mg/kg once daily for 3 days. The remaining group of animals was administered with test compounds intraperitoneally at a dose of 20 mg/kg. On fourth day, Pylorus was ligated as per the method of Shay *et al*.[17] Animals were fasted for 36 h before the pylorus ligation procedure. Four hours after the ligation, animals were sacrificed. The stomach was removed and opened along with the greater curvature. Ulcer index was determined by the method of Ganguly and Bhatnagar.[18]

## RESULTS AND DISCUSSION

### Chemistry

The key intermediate 5-fluoroimesatin (4) was prepared by reacting aniline (1) with chloral hydrate and hydroxylamine hydrochloride in sodium sulfate to give isonitrosoacetanilide (2), which was cyclized in the presence of concentrated sulfuric acid to afford the isatin (3). Compound 3 on reflux with 4-amino aniline in ethanol yielded the desired 4-fluoro-imesatin (4) in good yield (83%). The IR spectrum of compound (4) show intense peaks at 3220 cm-1 for amino (NH), 1675 cm -1 for carbonyl (C=O) stretching. 1H NMR spectra of compound (4) showed a multiplet at δ 7.15- 8.74 for aromatic (7H) protons and a singlet at δ 8.71 indicating the presence of NH. Data from the elemental analyses have been found to be in conformity with the assigned structure. Furthermore, the molecular ion recorded in the mass spectrum is also in agreement with the molecular weight of the compound. Data from the elemental analyses and molecular ion recorded in the mass spectra further confirmed the assigned structure.

The Schiff analogous of 3-(4-substituted amino)phenylimino)-5-fluoroindolin-2-one (**N_1_- N_10_**) were obtained by the condensation of amino group of 3-(4- amino) phenyl imino)-5-fluoroindolin-2-one (4) with a variety of alkyl and aryl ketones. The formation of title compounds [Scheme 1] is indicated by the disappearance of peak due to NH_2_ of the starting material in IR and ^1^H NMR spectrum [Table 2] of all the compounds (**N_1_-N_10_**). The IR and ^1^H NMR spectrum of these compounds showed the presence of peaks due to (N =CRR_1_) carbonyl (C=O), NH and aryl groups. The mass spectra of the title compounds are in conformity with the assigned structure. The mass spectrum of these compounds showed molecular ion peaks corresponding to their molecular formulae [Table 1]. A common peak at m/z 145 corresponding to indolin-2-one moiety appeared in all mass spectra of compounds (**N_1_-N_10_**). Elemental (**C, H, N**) analysis satisfactorily confirmed elemental composition and purity of the synthesized compounds.

### Pharmacology

Test for analgesic activity was performed by tail-flick technique using Wistar albino mice. The results of analgesic activity indicate that test compounds exhibited moderate analgesic activity at 30 min of reaction time; the activity increased at 1 h, further it reached to peak level at 2 h and declining in activity was observed at 3 h [Table 3]. Compound **N_3_** with 1-methyl butylidene substituents showed good activity among the aliphatic groups. Replacement of 1-methylbutylidene groups with alkyl chain with a cycloalkyl group and an aralkyl group (compounds **N_4_** and **N_5_** respectively) leads to moderate decrease in activity.

**Table 3:** Percent analgesic activity of synthesized compounds (tail-flick technique)

Compounds	Dose (mg/kg)	Percent analgesic activity			
		30 min	1h	2h	3h
N_1_	10	47 ± 1.25*	51 ± 1.32*	53 ± 1.15*	31 ± 1.96*
	20	54 ± 1.36**	58 ± 1.96**	60 ± 1.16***	47 ± 1.45*
N_2_	10	50 ± 1.25**	56 ± 1.32**	60 ± 1.15**	45 ± 1.68*
	20	57 ± 1.44**	60 ± 1.90**	64 ± 1.26***	50 ± 1.67*
N_3_	10	55 ± 1.75***	62 ± 1.82***	64 ± 1.35**	46 ± 1.96*
	20	62 ± 1.59***	66 ± 1.77***	69 ± 1.24***	52 ± 1.45*
N_4_	10	48 ± 1.28*	52 ± 1.52*	54 ± 1.15*	35 ± 1.76*
	20	55 ± 1.36*	57 ± 1.66**	59 ± 1.19**	44 ± 1.65*
N_5_	10	44 ± 1.76*	49 ± 1.88*	52 ± 1.55*	32 ± 1.26*
	20	52 ± 1.33*	55 ± 1.95**	59 ± 1.36**	42 ± 1.48*
N_6_	10	37 ± 1.87*	46 ± 1.42*	49 ± 1.15*	36 ± 1.96*
	20	49 ± 1.83*	54 ± 1.76**	59 ± 1.18**	43 ± 1.47*
N_7_	10	34 ± 1.74*	43 ± 1.72*	45 ± 1.45*	29 ± 1.69*
	20	47 ± 1.96**	52 ± 1.67**	56 ± 1.36**	44 ± 1.48*
N_8_	10	39 ± 1.75*	46 ± 1.43*	48 ± 1.24*	36 ± 1.36*
	20	48 ± 1.46*	51 ± 1.92**	54 ± 1.16*	39 ± 1.48*
N_9_	10	42 ± 1.82*	44 ± 1.72*	48 ± 1.59*	38 ± 1.72*
	20	46 ± 1.68*	51 ± 1.46**	57 ± 1.18**	45 ± 1.32*
N_10_	10	43 ± 1.55*	46 ± 1.72*	49 ± 1.52*	38 ± 1.92*
	20	48 ± 1.56*	52 ± 1.96**	58 ± 1.16**	42 ± 1.43*
Control		3 ± 0.35	5 ± 0.42	4 ± 0.50*	4 ± 1.56*
Diclofenac	10	37 ± 1.69*	43 ± 1.42*	45 ± 0.92*	33 ± 0.96*
	20	46 ± 0.95*	55 ± 1.16**	62 ± 1.49***	39 ± 1.13*

^a^Each value represents the mean ± SD (n = 6).

* Significance levels *P < 0.05

** *P*<0.01

*** *P*<0.001 as compared with the respective control

Replacement of aryl group at the N-4 position (compounds **N_6_, N_7_, and N_9_**) also results in decreasing activity. Placement of electron withdrawing group at N-4 aryl ring (compounds **N_7_, N_8_**) leads to further decrease of activity when compared to the reference standard diclofenac sodium.

Anti-inflammatory activity was evaluated by carrageenan-induced paw edema test in rats. The anti-inflammatory activity data [Table 4] indicated that all the test compounds protected rats from carrageenan-induced inflammation moderately at 30 min of reaction time; the activity increased at 1 h and it reached to peak level at 2 h. Declining in activity was observed at 3 h. Compound **N_3_** with 1-methyl butylidene substituents showed moderately more potent anti-inflammatory activity when compared to the reference standard diclofenac sodium. The compound **N_2_** with 1-methyl ethylidene substituents and compound **N_8_** showed equipotent anti-inflammatory activity when compared to the reference standard diclofenac sodium.

**Table 4:** Percent anti-inflammatory activity of synthesized compounds Percent anti-inflammatory activity of synthesized compounds (carrageenan-induced paw edema test in rats)

Paw edema test in rats).	Dose (mg/kg)	Percent anti-inflammatory activity			
		30 min	1h	2h	3h
N_1_	10	39 ± 1.25*	45 ± 1.32*	52 ± 1.15*	32 ± 1.96*
	20	44 ± 1.36**	52 ± 1.96**	58 ± 1.32**	37 ± 1.45*
N_2_	10	42 ± 1.25**	50 ± 1.38*	53 ± 1.15**	45 ± 1.68*
	20	51 ± 1.44**	57 ± 1.72**	62 ± 1.26**	54 ± 1.67*
N_3_	10	49 ± 1.75**	54 ± 1.82**	60 ± 1.35**	43 ± 1.96*
	20	54 ± 1.59***	62 ± 1.77***	66 ± 1.24***	52 ± 1.45*
N_4_	10	38 ± 1.82*	50 ± 1.24*	55 ± 1.15*	35 ± 1.76*
	20	45 ± 1.36*	56 ± 1.83**	60 ± 1.19**	44 ± 1.65*
N_5_	10	39 ± 1.56*	46 ± 1.88*	52 ± 1.65*	32 ± 1.26*
	20	45 ± 1.73*	54 ± 1.95**	59 ± 1.36**	42 ± 1.48*
N_6_	10	37 ± 1.67*	42 ± 1.42*	49 ± 1.58*	36 ± 1.96*
	20	46 ± 1.48*	55 ± 1.76**	59 ± 1.93**	38 ± 1.47*
N_7_	10	36 ± 1.74*	42 ± 1.72*	45 ± 1.55*	33 ± 1.69*
	20	44 ± 1.76**	55 ± 1.78**	58 ± 1.66**	41 ± 1.48*
N_8_	10	40 ± 1.65*	44 ± 1.53*	48 ± 1.74*	36 ± 1.36*
	20	49 ± 1.46*	52 ± 1.92**	56 ± 1.16*	39 ± 1.48*
N_9_	10	38 ± 1.82*	44 ± 1.32*	48 ± 1.99*	38 ± 1.72*
	20	46 ± 1.68*	51 ± 1.46**	57 ± 1.18**	45 ± 1.32*
N_10_	10	43 ± 1.55*	49 ± 1.72*	54 ± 1.52*	39 ± 1.92*
	20	48 ± 1.56*	54 ± 1.65**	58 ± 1.16**	42 ± 1.43*
Control		5.1 ± 0.35	6.1 ± 0.42	5.7 ± 0.30*	3.2 ± 1.56*
Diclofenac	10	32 ± 0.69*	38 ± 1.42*	39 ± 1.97*	33 ± 0.93*
	20	45 ± 1.65**	52 ± 0.96***	60 ± 1.52***	39 ± 1.13*

^a^Each value represents the mean ± SD (n = 6).

* Significance levels *P < 0.05

** *P*<0.01

*** *P*<0.001 as compared with the respective control

The ulcer index of the test compounds [Table 5] reveal that the compounds with open chain aliphatic substituents (compounds N_1_-N_5_) showed negligible ulcer index, whereas aryl substituents (compounds N_6_-N_9_) exhibited little increase in ulcer index and the aryl substituents containing electron withdrawing groups (compounds N_7_,N_8_, and N_10_) exhibited higher ulcer index over other test compounds. When compared to the reference standards aspirin (ulcer index 1.73 ± 0.41) and diclofenac (ulcer index 1.65 ± 0.59) the test compounds exhibited about 35-50% of the ulcer index of reference standards.

**Table 5:** Evaluation of the ulcerogenicity index

Compounds	Ulcer index
N_1_	0.84 ± 1.43
N_2_	0.97 ± 1.53
N_3_	0.85 ± 1.28
N_4_	0.89 ± 1.26
N_5_	0.82 ± 1.32
N_6_	0.84 ± 1.78
N_7_	0.78 ± 1.19
N_8_	0.92 ± 1.45
N_9_	0.75 ± 1.26
N_10_	0.81 ± 1.18			
Control	0.15 ± 0.24
Diclofenac	1.65 ± 0.48
Aspirin	1.73 ± 0.39

^a^Each value represents the means ± SD (n = 6). Significance levels, *P < 0.05 and **P < 0.01 as compared with the respective control.

## CONCLUSION

We have described the preparation of 3-(4- (benzylideneamino) phenylimino) 5-fluoro-indole-2-one. Several of these compounds have been evaluated as potential anti-inflammatory, analgesic agents with negligible ulcer index. In conclusion, this preliminary investigation showed that the presence of alkyl groups exhibited more analgesic and anti-inflammatory activities over aryl groups at the N-4 position. Among the synthesized compounds N_2_ and N_3_possessed the most prominent and consistent activity with maximum reduction of ulcerogenecity. Therefore, this series has opened new doors for possible modifications of the pharmacophoric requirements of NSAIDs and future exploitations.
